# Elastographic and ultrasound findings of the dog`s kidneys exposed to cigarette smoke

**DOI:** 10.29374/2527-2179.bjvm003525

**Published:** 2025-09-15

**Authors:** Samara Isis Rodrigues de Moraes, Giovanna Serpa Maciel Feliciano, Denise Jaques Ramos, Camila Silveira Stanquini, Diana Villa Verde Salazar, Luiz Paulo Nogueira Aires, Anna Carolina Mazeto Ercolin, Giovana Fumes Ghantous, Marcus Antônio Rossi Feliciano, Carlos Eduardo Ambrósio

**Affiliations:** 1 Faculdade de Zootecnia e Engenharia de Alimentos, Departamento de Medicina Veterinária, Universidade de São Paulo, Pirassununga, SP, Brazil.; 2 Departamento de Clínica e Cirurgia Veterinária, Faculdade de Ciências Agrárias e Veterinárias, Universidade Estadual Paulista, Jaboticabal, SP, Brazil.; 3 Faculdade de Zootecnia e Engenharia de Alimentos, Departamento de Ciências Básicas, Universidade de São Paulo, Pirassununga, SP, Brazil.

**Keywords:** kidney, elastography, dog, rim, elastografia, cão

## Abstract

This study evaluated the effects of exposure to tobacco smoke on the kidneys of household dogs using B-mode ultrasonography and Shear-wave Elastography (SWE). Fifteen adult dogs were analyzed and divided into two groups: one exposed to passive smoking for at least two years (n=7) and another non-exposed group (n=8), both without a history of kidney disease. The animals underwent clinical evaluation and laboratory tests, followed by B-mode ultrasonography and renal elastography. Blood test were normal for all individuals. Ultrasonography demonstrated a significant increase in renal echogenicity (p=0.0070, for both right and left kidneys) and irregular contours (p=0.0256 for the right kidney, and p=0.0070 for the left kidney) in the exposed group. The variables echotexture, corticomedullary ratio, and presence of alterations did not vary between the groups (p>0.05). Tissues were harder in exposed dogs (p=0.0492). These findings indicate that exposure to passive smoking may compromise canine kidney health, with early alterations detectable through ultrasonography and elastography. This study highlights the importance of raising awareness about the risks of smoking in domestic environments and underscores the need for further research to deepen the understanding of passive smoking’s impact on the renal morphology of domestic dogs.

## Resumo

Este estudo avaliou os efeitos da exposição à fumaça do tabaco sobre os rins de cães domésticos por meio de ultrassonografia em modo B e elastografia por ondas de cisalhamento (SWE). Quinze cães adultos, divididos em dois grupos: um exposto ao tabagismo passivo por pelo menos dois anos (n=7) e outro grupo não exposto (n=8), ambos sem histórico de doença renal. Os animais foram submetidos à avaliação clínica e exames laboratoriais, seguidos pela ultrassonografia e elastografia renal. Todos os indivíduos apresentaram exames de sangue dentro da normalidade. A ultrassonografia demonstrou aumento significativo da ecogenicidade renal (p=0.0070 para ambos os rins) e contornos irregulares (p=0.0256, rim direito e p=0.0070, rim esquerdo) no grupo exposto. Ecotextura, razão córtico-medular e presença de alterações não variaram entre os grupos (p>0.05). Houve maior rigidez tecidual em cães expostos (p=0.0492). Os achados indicam que a exposição ao tabagismo passivo pode comprometer a saúde renal canina, com alterações precoces detectáveis por ultrassonografia e elastografia. Este estudo destaca a importância de conscientizar sobre os riscos do tabagismo em ambientes domésticos e ressalta a necessidade de mais pesquisas para aprofundar a compreensão do seu impacto na morfologia renal de cães domésticos.

## Introduction

Active smoking is the leading cause of preventable death worldwide, accounting for approximately 8 million deaths annually, including those resulting from passive smoking (World Health Organization, 2023). Cigarette smoke contains more than 7,000 chemical substances, 70 of which are potentially carcinogenic (Organização Pan-Americana da Saúde, 2022). Studies indicate that smokers inhale only one-third of cigarette smoke, while the remainder disperses into the environment, exposing both people and animals to passive smoking (Agência Nacional de Vigilância Sanitária, 2020). The WHO (World Health Organization, 2023) warns that there are no safe levels of exposure to passive smoking and that only completely smoke-free environments ensure the prevention of health damage.

In Brazil, a significant portion of the adult and young population are smokers (Instituto Nacional de Câncer, 2025), and many households have pets (Instituto Brasileiro de Geografia e Estatística, 2020), suggesting that dogs and cats are also exposed to passive smoking. A study conducted by Milberger et al. (2009) showed that 63% of American households have a pet, and approximately one-fifth of pet owners are smokers.

Passive smoking presents an equivalent risk to active smoking for the development of cardiovascular diseases in humans, showing a strong association with hypertensive damage to target organs, with the kidney being one of the affected structures (Skipina et al., 2020). Additionally, a study by Jhee et al. (2019) demonstrated that exposure to side stream tobacco smoke is associated with a higher incidence of chronic kidney disease in humans. Similarly, Chen et al. (2021) found that exposure to passive smoking for two hours per week doubles the risk of developing kidney stones in humans.

Early diagnosis of kidney disease is essential, and laboratory tests combined with ultrasonography are fundamental tools for its detection (Bragato et al., 2017). Two-dimensional ultrasonography is widely used in veterinary medicine to assess dogs with chronic kidney disease, identifying structural renal alterations (Perondi et al., 2020). A study by Farhat et al. (2020) demonstrated that active smoking is associated with a reduction in renal dimensions in humans, highlighting the potential of ultrasonography in identifying smoking-related kidney damage.

Shear wave elastography is an innovative technique that quantifies tissue elasticity and is used to assess renal stiffness and identify early changes associated with chronic kidney disease (Cruz et al., 2021). This technique is based on the propagation of shear waves through the tissue, where the speed of these waves is directly proportional to the organ's stiffness (Comstock, 2011; Holdsworth et al., 2014). Thus, it allows a non-invasive evaluation of renal health, enabling early diagnoses.

Recent scientific evidence indicates that this technique is an efficient alternative for renal evaluation in humans with chronic kidney disease (Lim et al., 2021) and has already been applied in veterinary medicine for the analysis of different organs, such as the liver and kidneys (Facin et al., 2020). A study conducted by Cruz et al. (2021) suggests that ARFI elastography correlates tissue stiffness with chronic kidney disease stages. Additionally, elastography offers an advantage over conventional ultrasonography by providing a quantitative assessment of renal stiffness, aiding in monitoring the progression of kidney disease at different stages (Garcia et al., 2015).

Given this context, the objectives of this study were to evaluate the effects of passive smoking on the kidneys of household dogs through two-dimensional ultrasonography and shear wave elastography, describing their ultrasonographic and elastographic characteristics.

## Material and methods

### Selection and screening of animals

The project was approved by the Animal Ethics Committee (CEUA) of the Faculty of Animal Sciences and Food Engineering of the University of Sao Paulo (FZEA/USP), under the protocol number 5248010623.

The study was conducted at the Veterinary Hospital (HOVET) of FZEA/USP, and the animals belonged to owners from the city of the Institution. The owners were informed about the details of all stages of the project and signed a free and informed consent form, allowing their animals to participate in the study. Subsequently, a screening phase was carried out, consisting of anamnesis and selection of animals exposed to cigarette smoke, physical examination, complete blood count, and serum urea and creatinine measurement.

The control group consisted of 8 healthy adult dogs, including 3 males (1 neutered, and 2 intact) and 6 females (3 spayed, and 3 intact), ranging from 2 to 6 years old, and 9 to 35Kg. The body condition score was within normal limits (5-6) according to the Laflamme's scoring system (Laflamme, 1997). The control group was composed by Border Collie, Siberian Husky, Swiss Shepherd, Golden Retriever, Dachshund, Fila Brasileiro, and Australian Cattle Dog (one animal of each breed), and 2 mongreal dogs. All test results (physical and laboratory – complete blood count, urea, and creatinine levels) were within normal reference ranges, with no history of kidney disease and no exposure to tobacco smoke or significant environmental pollutants. The exposed group comprised 7 adult dogs, including 4 males (3 neutered, and 1 intact) and 3 females (1 spayed and 2 intact), ranging from 2 to 10 years old, and 14 to 53Kg. The body condition score was within normal limits (5-6) according to the Laflamme's scoring system (Laflamme, 1997). The exposed group consisted of French Bulldog, Border Collie, Siberian Husky, and Rottweiler (one dog of each breed), and 3 mongreal dogs. These animals had been exposed to cigarette smoke for at least 2 years, with no prior history of kidney disease and no exposure to significant environmental pollutants.

### B-Mode ultrasonographic analysis and shear wave elastography

The animals underwent an eight-hour solid fasting period before the ultrasonographic examination. Initially, a bilateral ventral abdominal trichotomy was performed, from the xiphoid process to the pelvic region, to improve renal structure visualization. The animals were then positioned in dorsal or lateral recumbency, with their heads facing the monitor, and a water-based conductive gel was applied for the ultrasound examination.

The ultrasound examinations were performed using the ESAOTE MX8® (Italy) ultrasound device. There was used a linear multi-frequency transducer (4-15 MHz) for elastography and a microconvex multi-frequency transducer (3-11 MHz) for B mode evaluations. First, a conventional B-mode ultrasound scan was performed following the abdominal scanning protocol, assessing kidney shape, echogenicity, homogeneity, parenchymal contour, corticomedullary ratio, and the presence of mineralization, cysts or signs of degeneration.

**Figure 1:**
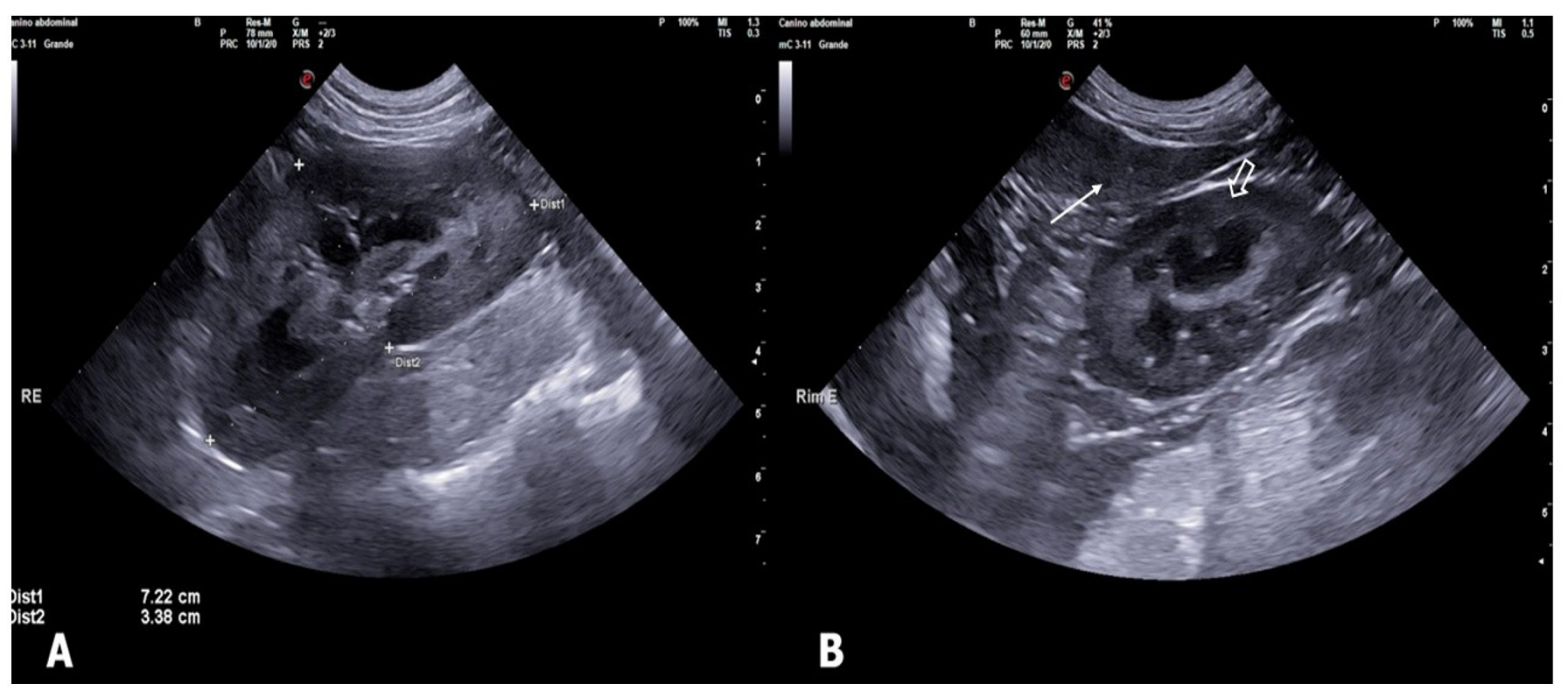
B-mode images of the left kidney. A) Left kidney in a longitudinal view, demonstrating the measurements of length (Dist 1) and height (Dist 2). B) Sagittal view of the left kidney, demonstrating the comparison of the echogenicity of the spleen (white arrow) and renal cortex (hollow arrow).

The renal echogenicity was evaluated by comparison with adjacent tissues. The left renal cortex should be hypoechoic to the splenic parenchyma, in healthy dogs. Cases when the left kidney was more echogenic than the spleen were classified as a hyperechoic kidney. The cortex of the right kidney was compared to the hepatic parenchyma and should be hypoechoic or isoechoic to the liver, in healthy dogs. A brighter right renal cortex would be classified as hyperechogenic in comparison to the liver (D’Anjou & Penninck, 2015) (Figure 1).

Renal cysts were detected as cavities with anechoic fluid, circumscribed by a thin, well-defined, echogenic capsule. Mineralizations were characterized by hyperechogenic structures of varied size, producing distal acoustic shadowing. The ultrasonographic appearance of degenerative processes can be variable, with increased cortical and/or medullary echogenicity, irregular contours and eventual ill-defined corticomedullary distinction.

Next, elastography evaluation was conducted using the same ultrasound device and transducer, with each B-mode image being analyzed through the activation of specific software (shear wave 2D, QelaXto™ 2D, ESAOTE) for quantitative tissue stiffness assessment.

**Figure 2:**
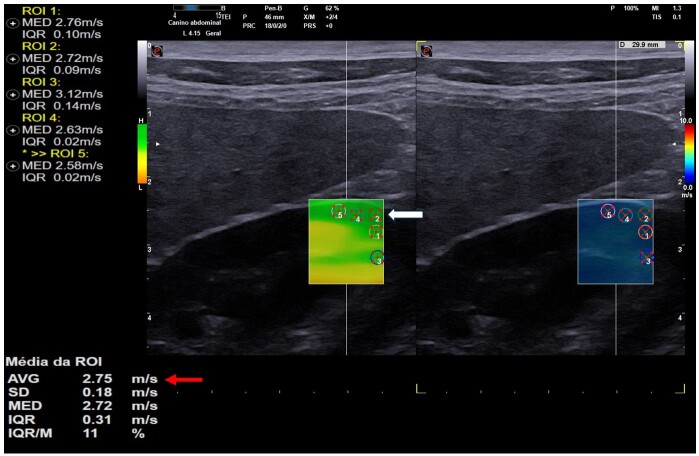
Quantitative elastography image of the left kidney of a 5-year-old mixed-breed dog. Measurements were performed exclusively in high-quality areas, indicated by the green color (white arrow), ensuring greater accuracy in analyzing the tissue shear wave propagation velocity, resulting in the average ROI (red arrow).

Images were captured in the longitudinal plane of each kidney, using a scale ranging from 0.0 to 10.0 m/s in all analyses. The quality map of the QelaXto™ 2D software was activated when the kidney was adequately framed in the image. In this analysis, areas represented in green indicated the highest image quality for obtaining quantitative elastography, while yellow represented medium quality, and orange/red indicated low quality. In all evaluated animals, quantitative elastography measurements were performed only in the high-quality regions, identified by the green color (Figure 2).

To assess tissue stiffness, five samples (regions of interest – ROIs) were randomly selected from different portions of the kidney, including both the cortex and medulla, using circles with a 0.2 cm diameter. The average of these five ROIs was calculated for each animal, resulting in a final mean value for everyone in both groups. The shear wave propagation velocity values of the analyzed portions were obtained and expressed in m/s (Figure 2).

The imaging results were analyzed using SAS software, version 9.4. Descriptive measures of count and percentage were calculated for categorical variables, while means and standard deviations were calculated for numerical variables. To evaluate the behavior of variables in the exposed and non-exposed groups, Fisher’s exact test was used for qualitative variables (Table 1). For quantitative variables, the Shapiro-Wilk test was conducted to assess normality, and independent samples Student’s t-tests were performed (Table 2). A significant level of 5% was adopted for all tests.

**Table 1:** Percentage of the ultrasonographic variables in the kidneys of the exposed and non-exposed dogs.

**Variable**	**Classification**	**Kidney**	**Exposed group n (%)**	**Non-exposed group n (%)**	**P value**
Echogenicity	Normoechogenic	Right	2 (28.57)	8 (100)	0.0070*
		Left	2 (28.57)	8 (100)	0.0070*
	Increased	Right	5 (71.43)	0 (0)	0.0070*
		Left	5 (71.43)	0 (0)	0.0070*
Echotexture	Homogeneous	Right	7 (100)	8 (100)	1
		Left	6 (85.71)	8 (100)	0.4667
	Heterogeneous	Right	0 (0)	0 (0)	1
		Left	1 (14.29)	0 (0)	0.4667
Parenchymal contour	Regular	Right	3 (42.86)	8 (100)	0.0256*
		Left	2 (28.57)	8 (100)	0.0070*
	Irregular	Right	4 (57.14)	0 (0)	0.0256*
		Left	5 (71.43)	0 (0)	0.0070*
Corticomedullary ratio	Preserved	Right	6 (85.71)	8 (100)	0.4667
		Left	7 (100)	8 (100)	1
	Poorly defined	Right	1 (14.29)	0 (0)	0.4667
		Left	0 (0)	0 (0)	1
Alterations found	Yes	Right	7 (100)	4 (50)	0.0769
		Left	7 (100)	4 (50)	0.0769
	No	Right	0 (0)	4 (50)	0.0769
		Left	0 (0)	4 (50)	0.0769

* P-value < 0.05

**Table 2:** Mean values of the region of interest from quantitative elastography for the exposed and non-exposed dogs.

**Variable**		**Exposed group**			**Non-exposed group**			**P-value**
		***n***	***M***	***SD***	***n***	***M***	***SD***	
	ROI’s mean	7	3.23	0.46	7	2.74	0.38	0.0492*

* P-value < 0.05;

n = number of animals; M = Mean; SD = Standard Deviation.

## Results

None of the 15 patients included in the study presented any abnormalities in physical evaluation and in the laboratory exams (blood count and serum concentration of urea and creatinine).

**Figure 3:**
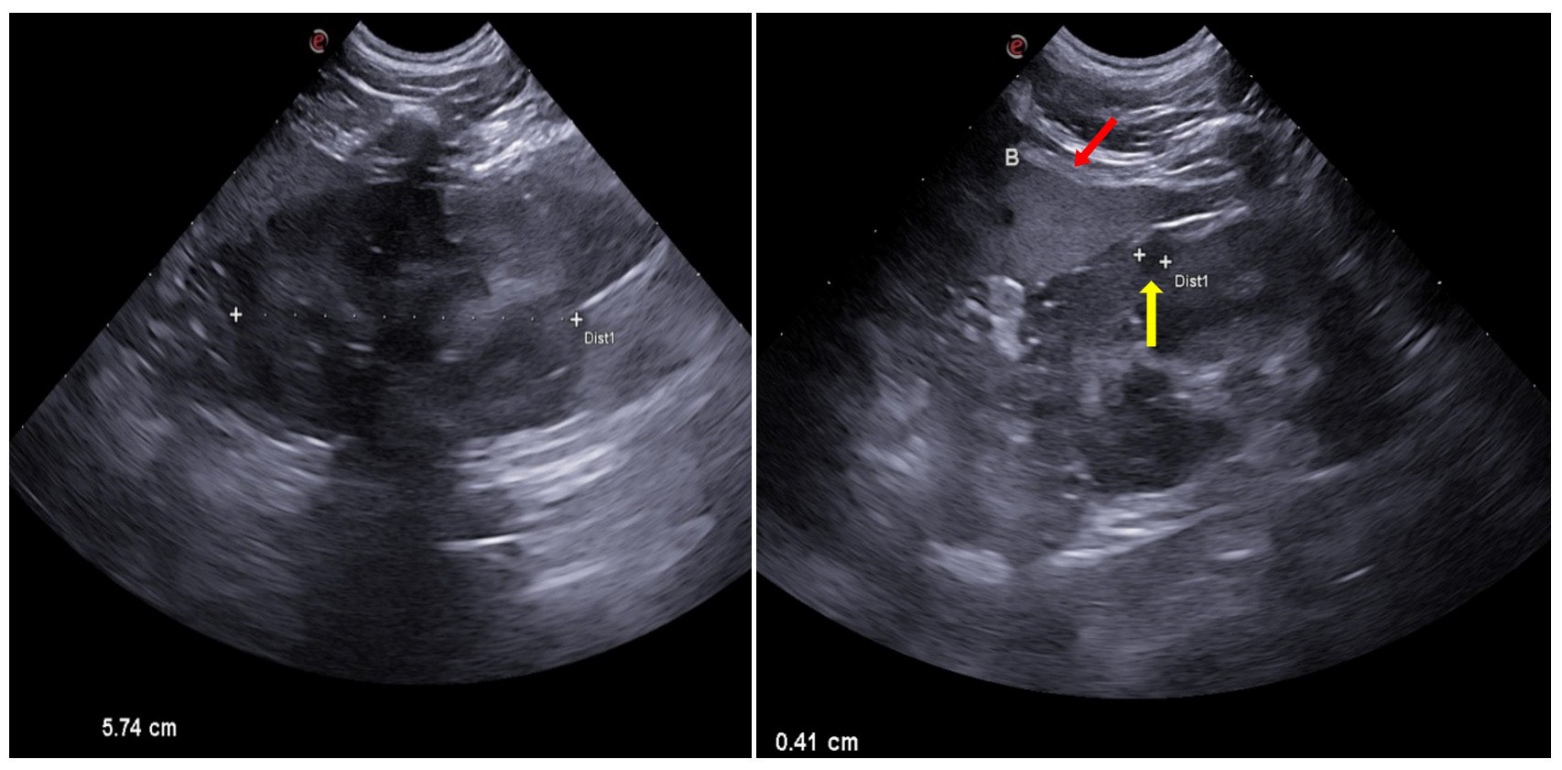
B-mode ultrasonography of the left kidney of a 6-year-old Border Collie from the exposed group. Irregular contours, increased echogenicity, and heterogeneous echotexture are observed, with the presence of a cystic area (yellow arrow) in the cortical region. The corticomedullary ratio is poorly defined, with an evident medullary signal, band sign, and mineralization in the pelvic recesses. In the background, the spleen can be seen (red arrow).

On B-mode ultrasound, no difference was found in echotexture (p=1 for the right kidney, and p=0.4667 for the left kidney), cortico-medullary ratio (p=0.4667 for the right kidney, and p=1 for the left kidney) and presence of kidney abnormalities (p=0.0769 for both right and left kidneys) (Figure 3) when comparing the control group to the exposed group (Table 1).

The qualitative variables of echogenicity (p = 0.0070 for both right and left kidneys) and parenchymal contour (p=0.0256 for the right kidney, and p=0.0070 for the left kidney) differed between the groups (Table 1). For both right and left kidneys, there was an increase in the echogenicity in the exposed group, while the control group presented normal echogenicity. As for the contours, most right and left kidney from the exposed group presented an irregular contour, while for the control group, contours were regular.

The results shown in Table 2 indicated a difference (p = 0.0492) in the ROI mean values from the exposed group when compared to the control group by means of Shear-wave Elastography (Figure 2).

In one patient from the exposed group, elastography assessment was not possible to perform due to the unusual deep position of the kidney, along with the size of the patient.

## Discussion

Two-dimensional ultrasonography is an essential technique for investigating renal conditions, enabling the identification of anatomical, functional, and structural changes (Bragato et al., 2017; Lang, 2010). An increase in renal echogenicity is associated with various pathologies in dogs, such as chronic kidney disease (Nyland et al., 2005; Perondi et al., 2020). In this context, all dogs in the control group (100%) presented normoechogenicity in both kidneys. On the other hand, 71.43% of the kidneys (left and right) evaluated in the exposed group showed increased echogenicity (Table 1). The increase in echogenicity may be associated with structural alterations such as fibrosis, sclerosis or infiltration (Buturović-Ponikvar & Visnar-Perovic, 2003). These findings suggest that exposure to passive smoke may be associated with changes in renal echogenicity, indicating a possible structural and/or physiological alteration. Changes in renal echogenicity were reported to occur even in the absence of creatinine alterations, such as reported in the present study. This can suggest that the increased echogenicity can be present at early stages of kidney disease (Koch et al., 2013).

Echotexture is one of the parameters evaluated in renal ultrasonography. A heterogeneous echotexture may be associated with alterations like renal dysplasia (Babicsak et al., 2012). In this study, as shown in Table 1, the echotexture was homogeneous in 100% of the kidneys in the unexposed dogs and in almost all the kidneys in the exposed dogs (100% right, and 85.71% left kidneys), with no statistical difference between the groups. Therefore, passive inhalation of cigarette smoke does not seem to interfere with this variable.

The renal parenchymal contour should be regular, with irregularities possibly linked to the presence of large renal cysts (Monteiro & Froes, 2009), neoplasms (Seyrek-Intas & Kramer, 2009) or infarction (Nyland et al., 2005). In the present study, 57.14% (right kidney) and 71.43% (left kidney) of the dogs in the exposed group showed irregular contours, while no such alteration was identified in the unexposed group (Table 1). This demonstrates a possible association between the evaluated smoke exposure and irregular renal contours.

On ultrasonographic examination, the difference between the cortical and medullary regions should be clear, and the renal pelvis may be visible (Nyland et al., 2005). Loss of definition of the corticomedullary junction was associated with various pathologies such as renal lymphoma, renal failure, renal dysplasia, and pyelonephritis, being an essential tool in evaluating renal integrity (Hünning et al., 2009; Notomi et al., 2006; Sousa et al., 2024). In this study, all kidneys (100%) from the control group, and almost all kidneys (85.71% right, and 100% left kidneys) from the exposed group showed a preserved corticomedullary junction, indicating maintenance of renal morphological architecture even with exposure to cigarette smoke (Table 1). Some authors pointed out that the degree of loss of the corticomedullary differentiation may be related to the stage of the chronic kidney disease (Bragato et al., 2017).

In the present study, alterations such as cortical cystic areas, mineralization of diverticula, and degeneration were observed. A total of 100% of the animals in the exposed group and 50% of the animals in the control group showed some type of ultrasonographic alteration (Table 1). Although the difference between the groups was not significant, the higher frequency of changes in the exposed group suggests a possible relationship with the exposure factor evaluated, highlighting the importance of further studies to better understand these findings.

Renal cysts can be located both in the cortex and in the renal medulla, and their origin can be congenital or acquired, usually resulting from conditions such as chronic kidney disease, glomerulonephritis, or renal dysplasia (Babicsak et al., 2012; Buturović-Ponikvar & Visnar-Perovic, 2003; Bragato et al., 2017). Mineralizations are associated with different pathological conditions, such as chronic renal failure, renal dysplasia, and osteosarcoma (Barazza et al., 2022; Hünning et al., 2009).

The degenerative process, is a condition of varying severity and can be caused by cardiac and hemodynamic alterations (Oliveira et al., 2019). Further studies, such as evaluating the systolic blood pressure of exposed animals, are needed to better clarify this association.

According to Nyland et al. (2005), no reliable ultrasonographic parameters are established to determine average renal size in dogs, due to the large variation in kidney size between dogs of similar weights, resulting from the different breeds. Some authors reported the use of kidney-to-aorta ratio to estimate renal size in the canine species (Kawalilak et al., 2019; Mareschal et al., 2007). The present study evaluated dogs of different sizes and breeds, and did not aimed to investigate whether the values found for renal size in the exposed and control groups were within normal limits for the different breeds.

Elastography detected an increase in the stiffness of the kidneys in dogs exposed to cigarette smoke, suggesting that this finding may be associated with structural changes resulting from this exposure. This result supports data from a previous study, which found that kidneys of dogs with chronic kidney disease (CKD) showed greater stiffness compared to normal tissues (Cruz et al., 2021). These authors used ARFI elastography and attributed the found alterations to pathophysiological processes such as fibrosis resulting from acute tubular necrosis. A study with bidimensional shear-wave elastography of different nephropathies showed higher stiffness in pathologic patients compared to the healthy dogs. However, this imaging modality did not differentiate between acute or chronic kidney disease (Puccinelli et al., 2023). Considering that passive smoking has already been associated with renal dysfunction in humans (Jhee et al., 2019), it is plausible that tobacco exposure may trigger similar mechanisms in dogs, contributing to the increase in renal stiffness observed in this study.

## Conclusion

The findings of this study indicate that exposure to passive smoke may be associated with renal changes in dogs, detectable by two-dimensional ultrasonography and shear wave elastography. The increased renal stiffness observed, along with increased echogenicity and irregularity of contours, suggests possible degenerative processes associated with the exposure. Despite the limitations, elastography demonstrated potential as a complementary diagnostic tool for early detection of renal abnormalities. Further studies with a larger and more homogeneous sample size and longitudinal follow-up are necessary to deepen the understanding of the effects of passive smoke on renal health in dogs. Additionally, the results highlight the importance of raising awareness about the risks of smoking in domestic environments, emphasizing the need to reduce passive exposure to minimize potential impacts on the health of dogs.
